# Chemical Derivatives of a Small Molecule Deubiquitinase Inhibitor Have Antiviral Activity against Several RNA Viruses

**DOI:** 10.1371/journal.pone.0094491

**Published:** 2014-04-10

**Authors:** Marta J. Gonzalez-Hernandez, Anupama Pal, Kofi E. Gyan, Marie-Eve Charbonneau, Hollis D. Showalter, Nicholas J. Donato, Mary O'Riordan, Christiane E. Wobus

**Affiliations:** 1 Department of Microbiology and Immunology, University of Michigan, Ann Arbor, Michigan, United States of America; 2 Department of Internal Medicine, University of Michigan, Ann Arbor, Michigan, United States of America; 3 Vahlteich Medicinal Chemistry Core, Department of Medicinal Chemistry, College of Pharmacy, University of Michigan, Ann Arbor, Michigan, United States of America; Centers for Disease Control and Prevention, United States of America

## Abstract

Most antiviral treatment options target the invading pathogen and unavoidably encounter loss of efficacy as the pathogen mutates to overcome replication restrictions. A good strategy for circumventing drug resistance, or for pathogens without treatment options, is to target host cell proteins that are utilized by viruses during infection. The small molecule WP1130 is a selective deubiquitinase inhibitor shown previously to successfully reduce replication of noroviruses and some other RNA viruses. In this study, we screened a library of 31 small molecule derivatives of WP1130 to identify compounds that retained the broad-spectrum antiviral activity of the parent compound *in vitro* but exhibited improved drug-like properties, particularly increased aqueous solubility. Seventeen compounds significantly reduced murine norovirus infection in murine macrophage RAW 264.7 cells, with four causing decreases in viral titers that were similar or slightly better than WP1130 (1.9 to 2.6 log scale). Antiviral activity was observed following pre-treatment and up to 1 hour postinfection in RAW 264.7 cells as well as in primary bone marrow-derived macrophages. Treatment of the human norovirus replicon system cell line with the same four compounds also decreased levels of Norwalk virus RNA. No significant cytotoxicity was observed at the working concentration of 5 µM for all compounds tested. In addition, the WP1130 derivatives maintained their broad-spectrum antiviral activity against other RNA viruses, Sindbis virus, LaCrosse virus, encephalomyocarditis virus, and Tulane virus. Thus, altering structural characteristics of WP1130 can maintain effective broad-spectrum antiviral activity while increasing aqueous solubility.

## Introduction

Effective treatment of infectious diseases is complicated by the pathogen's low genetic barrier for resistance, which inevitably results in drug escape when targeting pathogen-encoded proteins [1]–[3]. This has led to the emergence of pathogens that are highly resistant to most or all current antibiotics or antivirals [1], [2], [4], [5]. Targeting pathogen-encoded functions has one major advantage in that these drugs typically result in minimal side-effects in the host due to the high genetic differences between host and pathogen. However, to raise the barrier to developing drug resistance, new therapeutic strategies are necessary.

One way of circumventing drug-resistance lies in targeting host-encoded proteins, rather than the pathogen itself [6]–[8]. As host proteins are generally well conserved at their sequence level, as opposed to pathogen proteins, the effort required by the pathogen to alter its proteins enough to circumvent the absence of a crucial host factor is significantly higher. Thus, development of drug resistance while using host-targets is more difficult to achieve. More importantly, these host-targeted therapies could allow for treatment even when the infecting pathogen has not been fully identified, due to the fact that several different pathogens are redundant in their use of cellular proteins. Viruses often rely on overlapping host molecules or pathways for replication and survival [6], [8]. Recent evidence shows that many host requirements are seemingly conserved among similar viruses [9]–[12]. For example, non-immunosuppressive analogues of Cyclosporin A, a drug targeting cyclophilins in the host cell [13]–[15], can inhibit both HCV replication and HIV-1 [13], [16], [17]. In addition, targeting host-encoded proteins could also provide treatment options for pathogens that are obvious health threats but have no vaccine or treatment options. These include for example the non-segmented, negative-strand RNA viruses Nipah, Hendra and Ebola virus, and the positive-strand RNA genome-containing noroviruses. Therefore, therapies targeting host factors increase the possibility of affecting multiple pathogens and provide a strategy for the development of broad spectrum antimicrobials and/or antivirals.

Noroviruses are small non-enveloped viruses [18], of which human norovirus (HuNoV) accounts for most of the cases of sporadic and epidemic non-bacterial gastroenteritis worldwide in people of all ages [19]–[21]. Norovirus infections in the developed world are typically non-life threatening but are associated with tremendous economic costs [21], [22]. In contrast, high mortality rates are seen in the developing world with an estimated 200,000 children under the age of five succumbing to norovirus infections each year [21], [23]. However, there are currently no available vaccination or treatment strategies to prevent or control norovirus outbreaks. This is partly due to the absence of a reproducible culture system that permits the study of the complete viral life cycle and, until very recently, the lack of a small animal model for HuNoV [24], [25]. To study HuNoV replication, researchers use a replicon system stably expressing a plasmid containing the non-structural proteins of the prototypic norovirus strain, Norwalk virus [26], [27], and related culturable viruses as surrogates such as murine norovirus (MNV) [28] and Tulane virus [29]. MNV-1, the first culturable norovirus, replicates in macrophages and dendritic cells and shares many biological and molecular properties with HuNoV [28], [30], [31]. It is an enteric pathogen that is infectious after oral inoculation, replicates in the intestine and can be shed in the stool allowing for fecal-oral transmission [28]. It also shares the genomic organization, biophysical capsid properties and molecular mechanisms of translation initiation with HuNoV. Tulane virus is a genetically closely related calicivirus [32]. It infects B cells in the intestine of macaques [33] and the kidney cell line LLC-MK2 in tissue culture [29], causes diarrhea in its native host and, similar to HuNoV, recognizes human histo-blood group antigens (HBGAs) as an attachment receptor [32]–[34]. Both MNV and Tulane virus have the necessary tools of cell culture system, animal model, and reverse genetic system to make them promising model systems for the study of different aspects of human norovirus biology.

To address the lack of treatment options for norovirus infections, we decided to focus on targeting host-encoded functions rather than viral proteins due to the inherently high mutation rate of this RNA virus. We previously identified a partly selective small molecule deubiquitinase (DUB) inhibitor, WP1130, which exhibited potent antiviral effects against murine and human norovirus infection at the level of viral replication [35]. WP1130 did not affect MNV-1 entry. In addition to its anti-cancer properties [36], [37], WP1130 also reduced replication of several other RNA viruses, as well as bacteria [35], [38], which point to its potential as a broad-spectrum antimicrobial. WP1130 inhibits USP9X, USP5, USP14, UCH37, UCH-L1 in lymphoma cells, and potentially other DUBs as well [37]. The antiviral activity of WP1130 against MNV-1 is mediated in part through inhibition of USP14 [35]. Despite these promising antimicrobial features, WP1130 is not very soluble, making it a less-than-ideal candidate for *in vivo* experiments. Thus, our main goal was to identify compounds with improved drug-like features while maintaining broad-spectrum antiviral activity of WP1130 *in vitro*. A library of WP1130 derivatives was generated by addition of polar side chains and halogen modifications in pyridine functions off the double bond in the parent molecule. Screening of these derivatives would additionally allow the identification of active parts of the molecule with regards to their antiviral activity. We used a two-stage screening process. First, we identified the most effective anti-MNV-1 compounds by using concentration and treatment specifications previously identified with WP1130. The lead compounds were then tested in a second stage *in vitro* screen for minimal cellular toxicity and strong antiviral activity against a broader panel of RNA viruses. Here, we show that four small molecules derived from WP1130 are effective antivirals against MNV-1, Norwalk virus, La Crosse, Sindbis, Encephalomyocarditis virus, and Tulane virus. In addition, these broad spectrum antiviral compounds show increased solubility compared to the parent compound WP1130.

## Materials and Methods

### Cells, mice and viruses

RAW 264.7 cells, Vero cells and Be2-(c) cells were obtained from ATCC (Manassas, VA) and maintained as previously described [31], [35]. HG23 cells [27] containing the Norwalk virus replicon were obtained from Dr. Kyeong Chang (Kansas State University) and cultured as previously described [27]. LLC-MK2 cells [39] were obtained from Dr. Tibor Farkas (Cincinnati Children's Hospital Medical Center, Cincinnati, OH) and propagated as described for RAW 264.7 cells.

Swiss Webster mice were purchased from Charles River and housed at the University of Michigan in accordance with federal and university policies as outlined in the Guide for the Care and Use of Laboratory Animals and approved by the University of Michigan Committee on Use and Care of Animals. Bone marrow-derived macrophages (BMΦ) were isolated from Swiss-Webster mice as previously described [31].

The plaque purified MNV-1 clone (GV/MNV1/2002/USA) MNV-1.CW3 was propagated and used at passage 6 for all experiments [40]. Encephalomyocarditis virus, Sindbis virus, and La Crosse virus were obtained from Dr. David Miller (University of Michigan) and propagated in Vero cells or Be2-(c) cells as previously described [41]. Tulane virus was obtained from Dr. Tibor Farkas (Cincinnati Children's Hospital Medical Center, Cincinnati, OH) and propagated as previously described [29].

### Compounds

**Figure 1:**
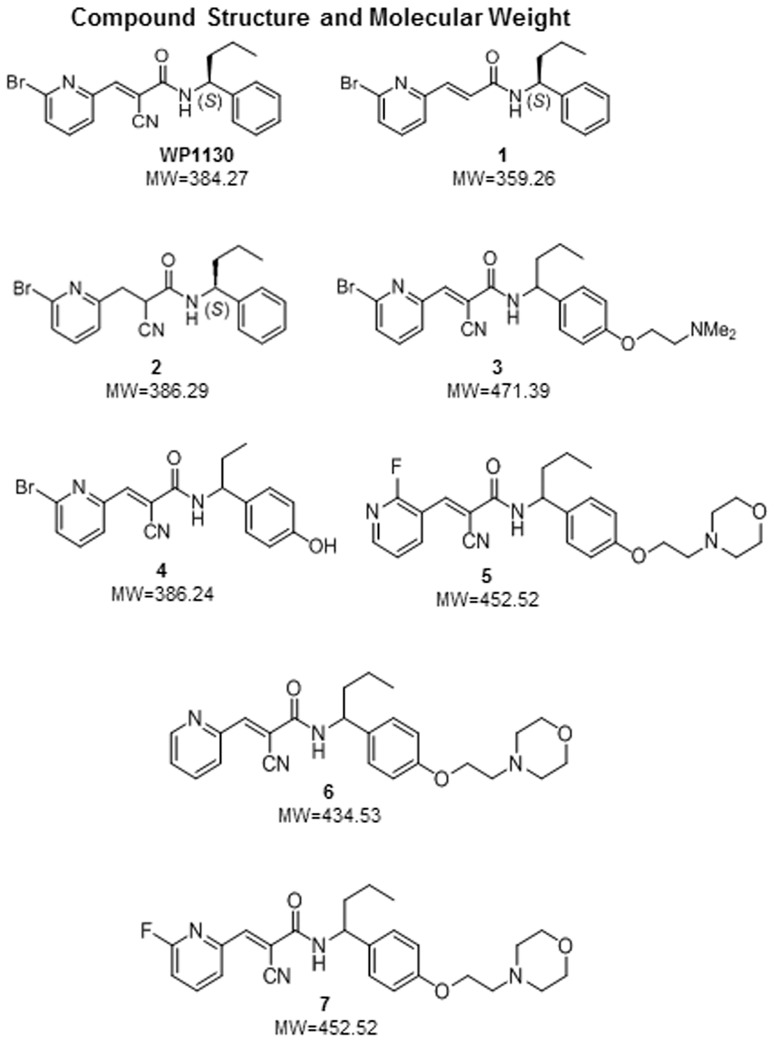
Molecular structures and weights for each of the compounds tested.

All small molecules were dissolved in DMSO (Sigma-Aldrich, St. Louis, MO), aliquoted and stored at −80°C. WP1130 and its derivatives 1–7 (Fig. 1) were synthesized by the Vahlteich Medicinal Chemistry Core (University of Michigan).

### Cellular toxicity and CC_50_ determination

All compounds were screened for cellular toxicity using the WST-1 assay (Roche, San Francisco, CA), which measures mitochondrial dehydrogenase activity. Assay conditions mimicked experimental conditions for infection. Briefly, cells were seeded at 5×10^4^ cells per well in 96-well plates, the night before the toxicity assay. Different concentrations of compounds or vehicle (dilution matched/equivalent volume), were added in duplicate in 90 µl of media, for 30 minutes at 37°C. Cells were then placed on ice for 1 hour (virus attachment simulation), after which they were washed three times with ice-cold PBS, and media containing compounds was added back. Cells were incubated for up to 8 hours at 37°C and 5% CO_2_. One hour prior to the end of the experiment, 10 µl of WST-1 was added to the cells. After that hour, samples were shaken for 1 minute, and OD_440_ and OD_600_ were determined. Results were calculated as % viability, and represent the mean of the absorbance values for each concentration divided by the mean of the absorbance values for untreated cells. These absorbance values were calculated using the formula Absorbance (A) at 440 nm–A 600 nm, after the background (WST-1 reagent alone) was subtracted. For CC_50_ determination, non-linear regression analysis was performed from the viability assay dilution curves using GraphPad Prism software v6.02 (La Jolla, CA).

### Virus infection, EC_50_ determination, and plaque assay

RAW 264.7 cells, BMΦ, Be-2c, Vero cells or LLC-MK2 cells were plated at 5×10^5^ cells per well in 12-well plates and allowed to attach overnight. The following day, cells were incubated with 5 µM of the indicated compound for 30 minutes at 37°C. Cells were infected with the specified virus at an MOI of 5 for 1 hour on ice. Virus inoculum was removed by washing cells three times with ice-cold PBS. Media containing the appropriate compounds was added back to cells, and the infection was allowed to proceed for either 8 or 10 hours (MNV-1 in RAW 264.7 or BMΦ, respectively), 12 hours (Sindbis virus and Encephalomyocarditis virus in Vero cells, La Crosse virus in Be2-(c) cells), or 24 hours (Tulane virus in LLC-MK2 cells). Cells were freeze-thawed twice. Viral titers were determined by plaque assay as previously described on RAW 264.7 cells for MNV-1 [31], on LLC-MK2 cells for Tulane [29], or on Vero cells for all other viruses [35]. Compound EC_50_ values were determined from plaque assay results or qRT-PCR (for Norwalk virus as described below), after cells were pre-treated with varying concentrations of compounds, using non-linear regression analysis in the GraphPad Prism software v6.02 (La Jolla, CA).

### HuNoV replicon assay

HG23 cells containing the Norwalk virus replicon plasmid under G418 selection were plated at 5×10^5^ cells per well in 12-well plates and allowed to attach overnight. Media was replaced with one containing either DMSO or the compounds of interest. After 24 hours, cells were washed once with PBS and total cellular RNA was isolated using the QIAGEN RNeasy Mini kit (QIAGEN, Valencia, CA). RNA was subjected to on-column RNase-free DNase treatment (QIAGEN, Valencia, CA) for 15 minutes at room temperature following the manufacturer's protocol. After elution, another round of DNase treatment was performed using the DNA-free DNase removal kit (Ambion, Austin, TX) following the manufacturer's protocol. RNA concentration and purity were measured using a spectrophotometer, calculating the 260/280 ratio. RNA integrity (as well as the absence of DNA contamination) was confirmed by One-Step Reverse Transcription-Polymerase Chain Reaction (RT-PCR) using “No RT” controls. If DNA contamination was detected, another round of DNase treatment was performed. Norwalk virus genomes were then quantitated by qRT-PCR as previously described [27].

### Deubiquitinase Activity Assay

2×10^6^ RAW 264.7 cells were grown for 24 h in 60 mm dishes followed by treatment with vehicle or 5 µM of WP1130 or compound **7**. After 4 h of treatment, the cells were collected and lysed in ice-cold deubiquitinase buffer containing 50 mM Tris–HCl, pH 7.5, 0.5% NP-40, 5 mM MgCl_2_, 150 mM NaCl and 1 mM freshly added phenylmethylsulfonylfluoride. Twenty µg of clarified lysates from untreated, WP1130-, or compound **7**-treated cells were incubated with 0.2 µM of HA-ubiquitin labeled vinyl sulfone (HA-ubVS) for 75 min at 37°C. The reaction was stopped by adding SDS Laemmli sample buffer. Lysates were run on 6–12% SDS PAGE gradient gels and subjected to immunoblot analysis. The DUBs bound to ubiquitin-vinyl sulfone were detected by immunoblotting with anti-HA antibody.

## Results

### Identification of WP1130-derivatives with anti-norovirus activity

**Figure 2:**
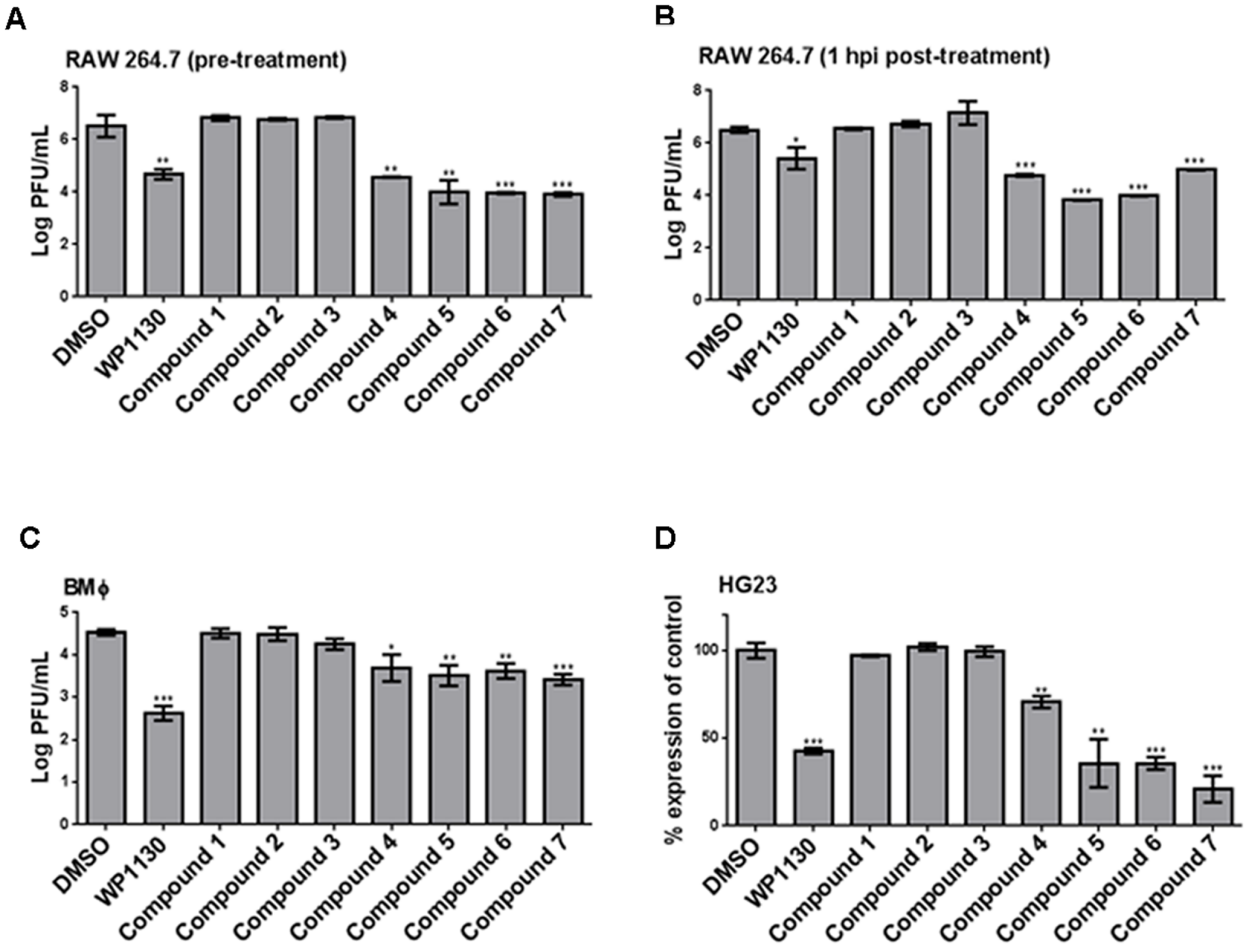
WP1130-derivatives inhibit norovirus replication. (A and B) RAW 264.7 cells or (C) Swiss Webster bone marrow-derived macrophages (BMΦ) were incubated with DMSO (volume-matched) or 5 µM of WP1130 or seven WP1130-derivatives (compound 1–7), for 30 minutes prior to infection (A and C) or 1 hour post-infection (B). Cells were infected with MNV-1 (MOI 5) in the presence of compounds or DMSO, for 8 hours (RAW 264.7) or 10 hours (BMΦ). Virus titers were determined by plaque assay. (D) Norwalk virus replicon-bearing HG23 cells were treated with DMSO (volume-matched) or 5 µM of the indicated compound for 24 hours, after which Norwalk virus genomes were quantitated by qRT-PCR. Norwalk virus genome copy number was normalized to DMSO-treated samples. Data from three to five independent experiments are presented as means, with error bars indicating SEM of the results. Significance was calculated by comparing each treatment to vehicle, using a Student's T test. Significant results are indicated (*P<0.05, **P<0.01 and ***P<0.001).

To identify molecules derived from WP1130 that retained the broad-spectrum antiviral activity *in vitro* but showed improved drug-like features, we screened a library of 31 compounds synthesized at the University of Michigan's Vahlteich Medicinal Chemistry Core. Figure 1 depicts the structures of four effective compounds used in this study, as well as three inactive derivatives, and the parent compound WP1130. We first screened for antiviral activity against MNV-1 in RAW 264.7 cells at a concentration of 5 µM, following the protocol previously described for WP1130 [35]. Following a 30 minute pre-treatment, 17 compounds with moderate to high anti-MNV-1 activity were identified (data not shown). Of these, four showed similar or greater decreases in viral titers when compared to WP1130, and were selected for further analyses (Fig. 2A, compounds **4**–**7**). Fourteen compounds had no significant effect on MNV-1 replication (Fig. 2A, compounds **1**–**3**, and data not shown). Pre-treatment of RAW 264.7 cells with WP1130 results in an average 2.0-log decrease in MNV-1 titers as compared to the DMSO vehicle control (Fig. 2A). Compound **4** (abbreviated **4**) exhibited a similar effect as WP1130 in reducing MNV-1 titers (1.95-log decrease compared to control). Compounds **5**, **6** and **7** each exhibited a more pronounced decrease in viral titers when administered before infection (2.52-log, 2.55-log and 2.60-log decrease in viral titers, respectively, compared to DMSO; Fig. 2A) with compounds **6** and **7** reaching statistical significance (t test, P<0.01).

To assess the effective duration of antiviral activity, time-of-addition studies were performed by adding compounds **4**–**7** up to 4 hours post infection. All four compounds retained antiviral activity when administered 1 hour post infection (Fig. 2B) with viral titer decreases of 1.73-log (compound **4**), 2.67-log (compound **5**), 2.50-log (compound **6**), and 1.51-log (compound **7**), when compared to DMSO. All compounds were similarly or more effective than WP1130 at this time point (Fig. 2B), with compounds **5** and **6** showing statistically significant differences in their antiviral activity compared to WP1130 (t test, P<0.01). However, no antiviral activity was observed when compounds **4**–**7** were administered 2–4 hours post-infection (data not shown).

To determine whether antiviral activity was retained in primary cells, primary bone marrow-derived murine macrophages were pre-treated with compounds **1**–**7** (Fig. 2C). Decreases in MNV-1 titers ranging from 0.84-log to 1.12-log were observed compared to vehicle control, demonstrating the antiviral activity was not limited to a tissue-culture adapted cell line. However, the antiviral activity of compounds **4–7** in primary macrophages was significantly less compared to WP1130.

As WP1130 also reduced replication of the human norovirus Norwalk virus in replicon-bearing cells [35], we next assessed if the WP1130 derivatives **1**–**7** also had an effect against this human pathogen (Fig. 2D). Norwalk virus replicon-bearing hepatoma cells (HG23) were grown in the presence of each compound or DMSO for 24 hours, after which Norwalk virus genomes were quantitated using qRT-PCR as previously described [27]. The same four compounds with anti-MNV-1 activity also significantly reduced the level of detectable Norwalk virus genomes. Compound **4** reduced genome levels by approximately 30%, whereas compounds **5** and **6** both reduced levels by about 64%. Compound **7** caused the highest reduction with an approximate 80% decrease in detectable viral genomes when compared to DMSO vehicle, and the reduction was more significant than that caused by WP1130 (t test, P<0.05). Reduction in viral genomes only occurred if HG23 cells were treated with the compound for at least 24 hours but not with 8 or 12 hour treatments (Fig. 2D, and data not shown). As a positive control HG23 cells were treated with ribavirin, a nucleoside analogue known to reduce Norwalk virus replication [26], which diminished viral RNA levels to 10–20% of the vehicle control-treated cells (PBS for Ribavirin, data not shown).

These results demonstrate that at 5 µM of compounds **4**–**7** significantly inhibited both MNV-1 infection and Norwalk virus replication *in vitro*, when added up to 1 hour post-infection for MNV-1 or for at least 24 hours in Norwalk virus replicon-bearing cells.

### In vitro toxicity of WP1130-derivatives and determination of CC_50_ and EC_50_ values

**Figure 3:**
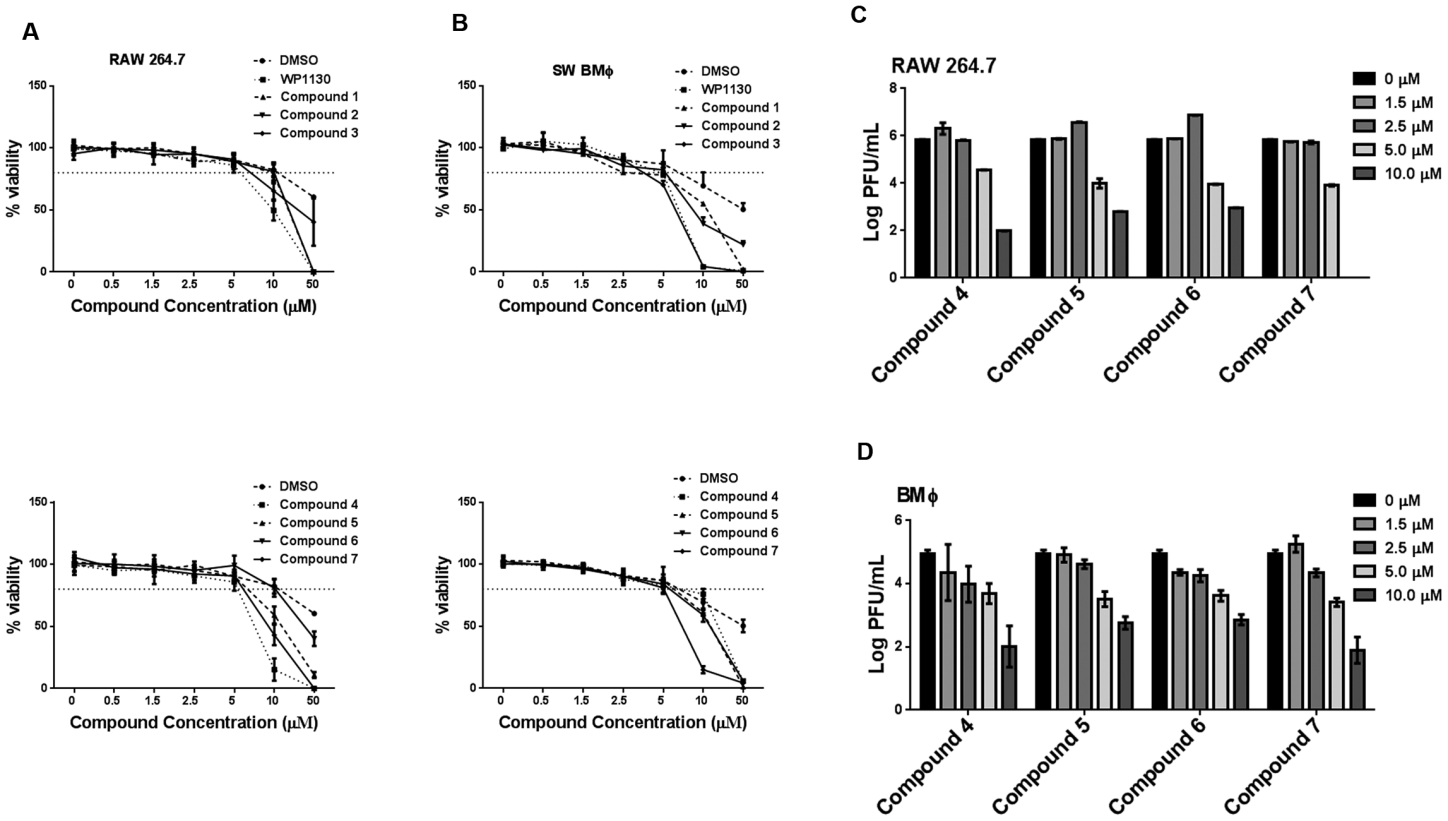
WP1130-derivatives are effective against MNV-1 with minimal toxicity at working concentration. (A) RAW 264.7 cells (top and bottom), or (B) Swiss Webster bone marrow-derived macrophages (BMΦ, top and bottom) were incubated for 30 minutes with DMSO (volume-matched) or indicated concentrations of WP1130 or WP1130-derivatives (compounds 1–7). After incubation, cells were subjected to conditions that mimicked the virus infection protocol. WST-1 assays for cell viability were then performed and data was plotted as percent viability compared to untreated cells. Data are presented as a mean of three independent experiments. (C) RAW 264.7 and (D) BMΦ cells were treated for 30 minutes with different concentrations of active compounds 4–7, and then infected with MNV-1 (MOI 5) for 8 or 10 hours, respectively. Viral titers were determined by plaque assay. Data from three to five independent experiments are presented as means, with error bars indicating SEM of the results.

In order to verify that the antiviral effect observed with the WP1130-derivatives was not due to cellular toxicity, we performed viability assays on RAW 264.7 cells and bone marrow-derived macrophages treated with the compounds at different concentrations (Fig. 3A and B) and calculated cellular cytotoxicity 50 (CC_50_) values. The CC_50_ value is the concentration of chemical agent, which is lethal to 50% of the cells at a given exposure time and experimental conditions. Similar to the parent compound WP1130, RAW 264.7 macrophages that received compounds **1**–**7** showed no significant difference in viability compared to the DMSO control at concentrations of 5 µM or lower (Fig. 3A). Cell viability remained at or above 80% up to 5 µM and then gradually declined. In bone marrow-derived macrophages (Fig. 3B), cell viability also remained above 80% at our working concentration of 5 µM. Table 1 depicts CC_50_ values for all compounds, further demonstrating suitable viability during our experiments. However, none of the compounds exhibited decreased toxicity when compared to WP1130 (Fig. 3A and B).

**Table 1:** 

Compound	Solubility (µM)^a^	RAW 264.7		SW BMΦ		HG23	
		CC_50_ (µM)^b^	EC_50_ (µM)^c^	CC_50_ (µM)	EC_50_ (µM)	CC_50_ (µM)	EC_50_ (µM)
WP1130	3.2	9.95	N/A	6.46	N/A	6.20	N/A
Compound 1	n.d.^d^	24.73	N/A^e^	10.42	N/A	7.20	N/A
Compound 2	n.d.	24.94	N/A	8.53	N/A	8.71	N/A
Compound 3	19	9.43	N/A	6.12	N/A	7.82	N/A
Compound 4	n.d.	11.01	5.21	15.23	6.46	9.26	6.52
Compound 5	14	15.44	4.94	11.31	5.24	7.61	4.28
Compound 6	160	7.46	4.91	11.11	3.95	10.42	4.17
Compound 7	14	13.65	5.59	7.11	5.3	6.12	3.37

a Solubility was calculated as per Lipinski et al. [44].

b CC_50_ and EC_50_ values were calculated using viability and viral titer/genomes data, respectively, as obtained through WST-1 or plaque assays of cells treated with varying concentrations of compounds and then infected with MNV-1 or containing the Norwalk replicon. Values were extrapolated after non-linear regression.

c Only compounds with antiviral activity were used for EC_50_ determination.

d n.d.  =  not determined.

e N/A  =  not applicable.

We next determined the EC_50_ (effective concentration at which viral titers/genomes are reduced by 50%) for WP1130 derivatives (Table 1). As WP1130 showed marked toxicity at concentrations above 6 µM for all cell types tested, we did not perform a full EC_50_ evaluation on this compound. We previously determined the EC_50_ in RAW 264.7 cells to be 5.06 and similar values are estimated for HG23 and primary bone marrow-derived macrophages. RAW 264.7 or primary bone marrow-derived macrophages were either left untreated or pre-treated for 30 minutes with each of the four effective compounds (compounds **4**, **5**, **6**, and **7**) at concentrations ranging from 1.5 to 10 µM prior to infection with MNV-1 (Fig. 3C and D). At the 10 µM concentration, each of the four compounds showed the greatest decrease in viral titers. However, cell viability at or above 80% was only maintained at 5 µM. As indicated in Table 1, our working concentration of 5 µM is very close to the estimated EC_50_ values in both RAW 264.7 cells and murine primary macrophages.

**Figure 4:**
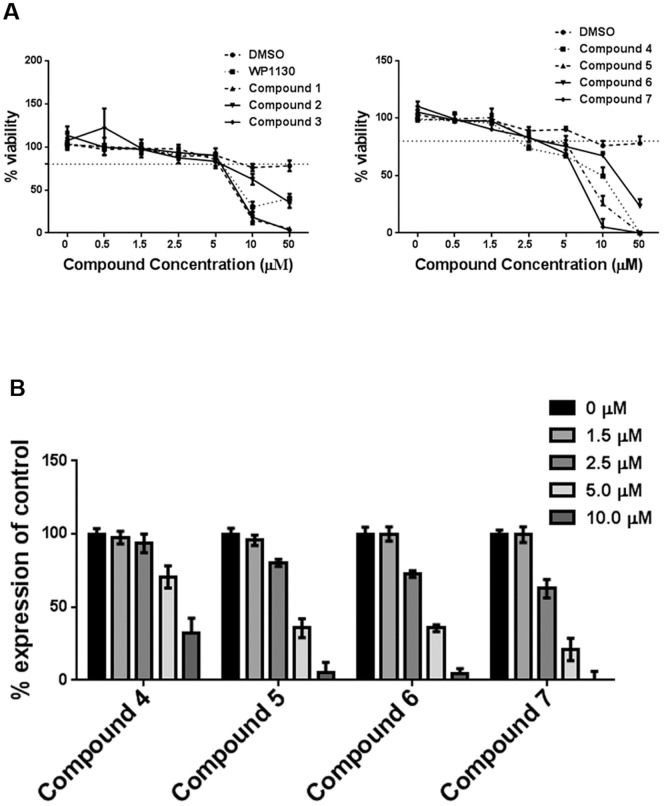
WP1130-derivatives are effective against Norwalk virus with minimal toxicity at working concentration. (A) HG23 Norwalk virus replicon-bearing cells were incubated with DMSO (volume-matched) or different concentrations of WP1130 and WP1130-derivatives (compounds 1–7) for 24 hours. WST-1 assays for cell viability were performed and data was plotted as percent viability compared to untreated cells. Data are presented as a mean of three independent experiments. (B) HG23-replicon cells were treated with the indicated concentrations of active compounds 1–4 for 24 hours after which Norwalk virus genomes were quantitated by qRT-PCR. Norwalk virus genome copy number was normalized to untreated samples. Data from three independent experiments are presented as means, with error bars indicating SEM of the results.

Similar viability analyses were performed on HG23 Norwalk virus replicon-bearing cells by incubating cells with compounds for 24 hours. At the effective concentration of 5 µM, cells retained approximately 80% viability (Fig. 4A and Table 1). EC_50_ calculations demonstrated that the working concentration of 5 µM was near the EC_50_ of these compounds (Fig. 4B and Table 1). Shorter incubation times of 8 and 12 hours with the compounds at 5 µM showed increased viability, but did not result in significant reductions in viral genome copies (data not shown).

Overall, these data confirm the antiviral activity of compounds **4**–**7** and demonstrate that 80% cellular viability is maintained at our working concentration of 5 µM.

### WP1130-derivatives have broad-spectrum antiviral activity

**Figure 5:**
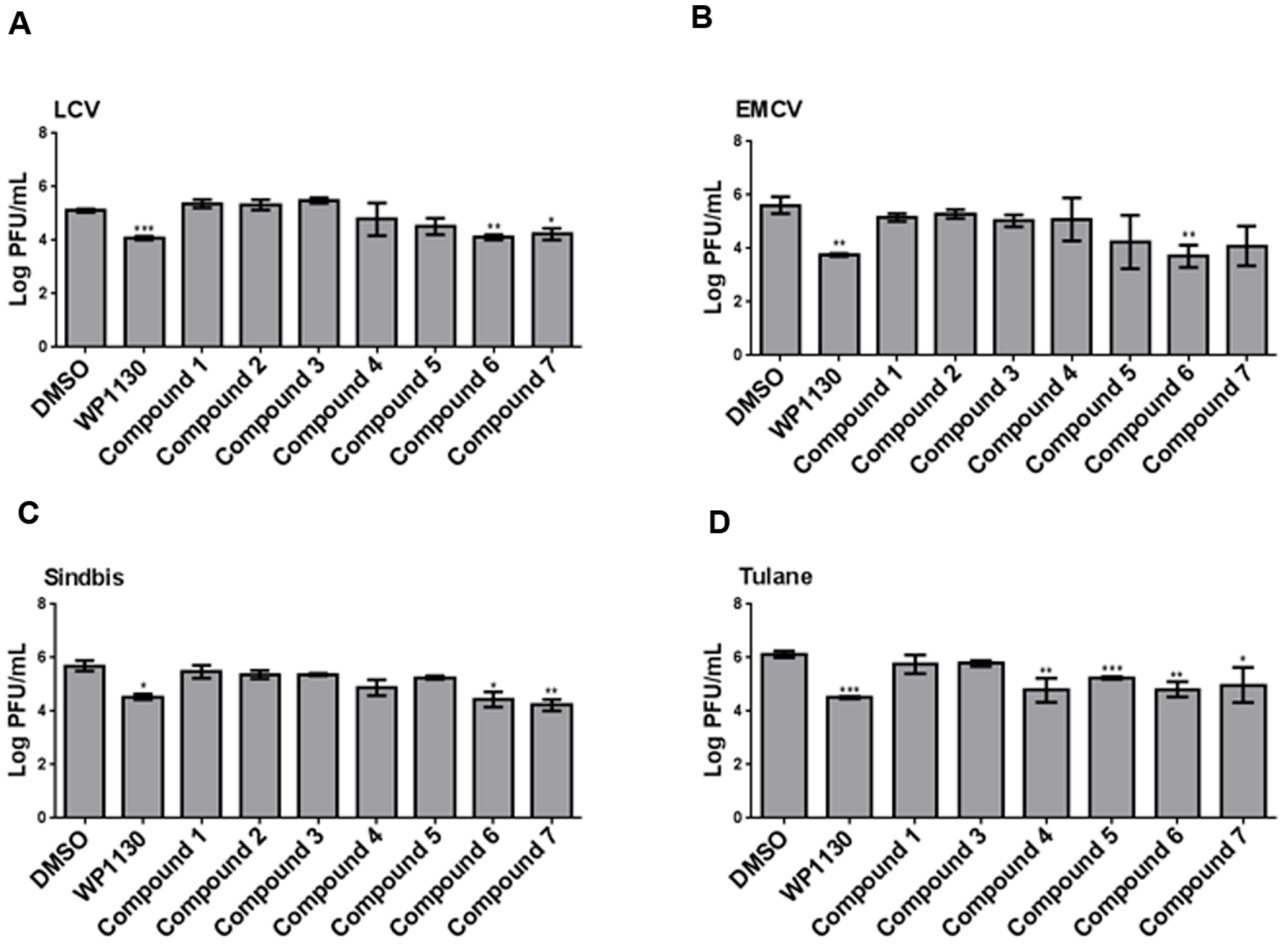
WP1130-derivatives show broad spectrum antiviral activity. (A–D) Cells were treated for 30 minutes with DMSO (volume-matched) or 5 µM of WP1130 or its derivatives prior to infection. (A) Treated Be2-c cells were infected with La Crosse virus (LCV, MOI 5) for 12 hours, and viral titers were determined by plaque assay on Vero cells. (B) Treated Vero cells were infected with Encephalomyocarditis virus (EMCV, MOI 5) for 12 hours, and viral titers were determined by plaque assay on Vero cells. (C) Treated Vero cells were infected with Sindbis virus (MOI 5) for 12 hours, and viral titers were determined by plaque assay on Vero cells. (D) Treated LLC-MK cells were infected with Tulane virus (MOI 5) for 24 hours, and viral titers were determined by plaque assay on LLC-MK cells. Data shown are representative of three independent experiments and are presented as means, with error bars indicating SEM of the results. Significance was calculated by comparing each treatment to vehicle, using a Student's T test. Significant results are indicated (*P<0.05, **P<0.01 and ***P<0.001).

WP1130 has broad spectrum antimicrobial efficacy [35], [38]. To examine whether the WP1130-derivatives maintained the broad spectrum antiviral activity *in vitro*, we next tested the antiviral effect of the WP1130-derivatives **1**–**7** on a group of RNA viruses with positive- or negative-sense RNA genomes, and with or without enveloped particles. Be2-c cells (Fig. 5A), Vero cells (Fig. 5B and C) or LLC-MK2 cells (Fig. 5D) were treated with DMSO or compounds **1**–**7** at 5 µM for 30 minutes prior to infection with either La Crosse virus (LCV), an enveloped, negative-sense RNA virus (Fig. 5A), Encephalomyocarditis virus (EMCV), a non-enveloped, positive-sense RNA virus (Fig. 5B), Sindbis virus, an enveloped, positive-sense RNA virus (Fig. 5C), or Tulane virus, a non-enveloped, positive-sense RNA virus (Fig. 5D). Similar to its parent compound WP1130, compound **6** significantly reduced infectious titers for all four viruses. Compound **7** significantly decreased infection of LCV, Sindbis and Tulane virus, but not of EMCV, although a trend towards antiviral activity was present as well. Compounds **4** and **5** were only effective against Tulane virus. The working concentration of 5 µM for all compounds used in these studies were non-toxic (i.e., cell viability at or above 80%) in each of the cell types (data not shown). Infection with Vesicular Stomatitis Virus (VSV), a negative-sense, enveloped virus, or rhinovirus (RV39), a positive-sense, enveloped virus, was not inhibited by WP1130 or compounds **1**–**7** (data not shown). Taken together, these data demonstrate the broad spectrum antiviral activity of specific WP1130-derivatives *in vitro*.

### A WP1130-derivative retains DUB inhibitory activity in vitro

**Figure 6:**
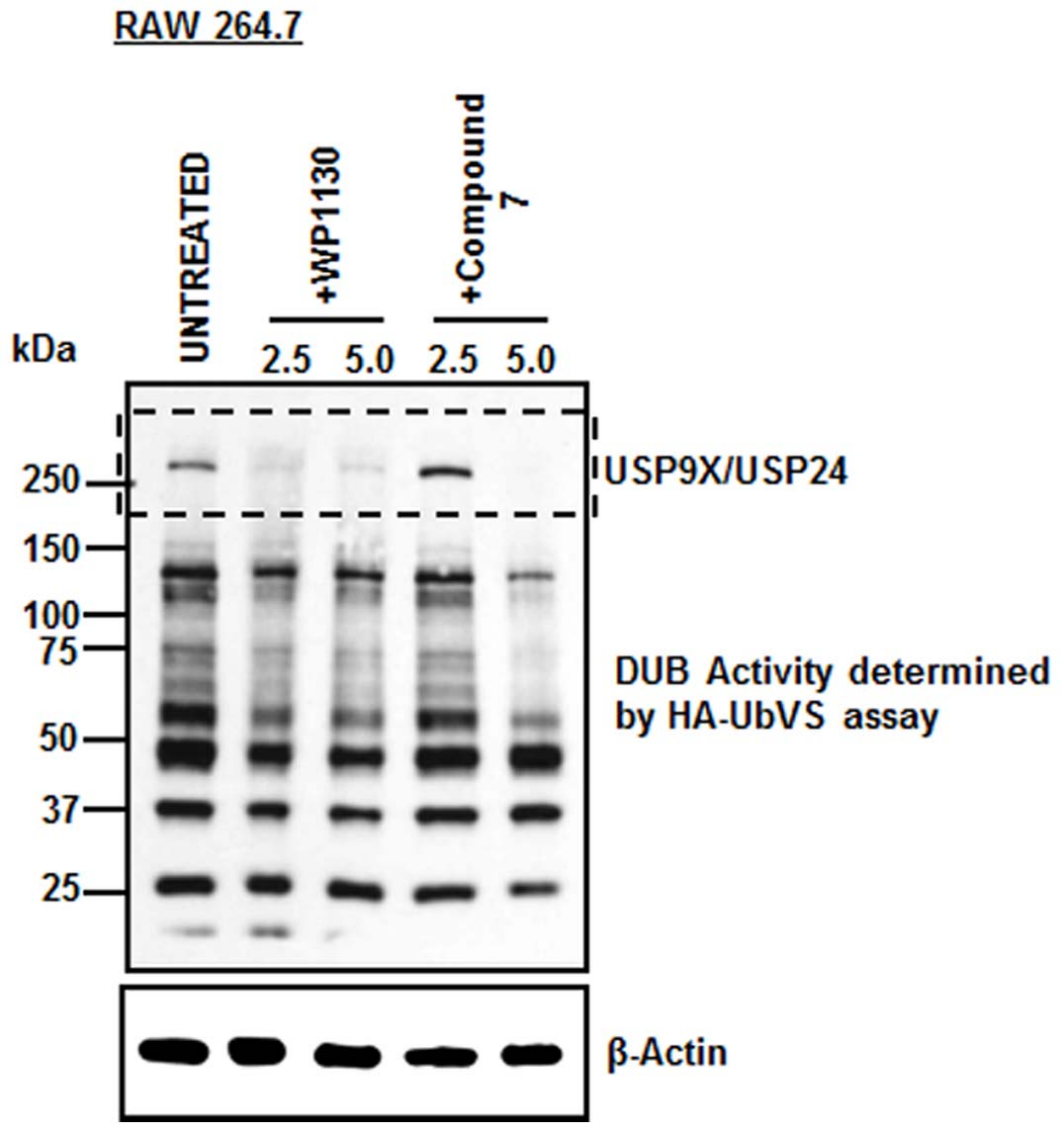
Inhibition of deubiquitinating enzyme activity by WP1130 and its derivative in RAW 264.7 cells : RAW 264.7 cells treated with vehicle alone or the indicated concentration of WP1130 or compound 7 for 4 h were lysed and labeled with HA-Ub-Vinyl sulfone substrate. The bound deubiquitinase enzymes were detected by immunoblot analysis with anti-HA antibody.

As WP1130 can directly inhibit DUB activity in intact cells [35], [37], we next wondered whether its derivatives retained this activity in RAW 264.7 cells. Unfortunately, we only had sufficient quantities of WP1130 and compound **7** to perform DUB inhibition assays. We used a previously described technique [42] to identify the profile of active cellular DUBs in intact cells. This technique involves the use of an HA-labeled ubiquitin substrate conjugated to a vinyl sulfone group (HA-UbVS), which acts as a DUB suicide substrate, forming a covalent adduct with active DUB enzymes. A profile of active DUBs are then identified by immunoblotting with anti-HA antibody. RAW 264.7 cells treated with WP1130 and compound **7** showed diminished expression of active DUBs as evident by the reduction in HA-labeling (less intense banding patterns), when compared to untreated cells (Fig. 6). Particularly striking is the inhibition of DUBs clustered in the higher molecular weight region, which typically correspond to USP9x and USP24. These data demonstrate that compound **7** retained the selective DUB inhibitory activity observed with WP1130.

## Discussion

Herein, we screened a small molecule library of derivatives of the selective DUB inhibitor WP1130 with known broad spectrum antiviral activity against a panel of RNA viruses *in vitro*. We identified small molecule derivatives of WP1130 with *in vitro* antiviral activity against MNV-1, the HuNoV Norwalk virus, the related calicivirus Tulane virus, the bunyavirus La Crosse virus, the alphavirus Sindbis virus, and the picornavirus Encephalomyocarditis virus. No antiviral activity was observed towards the rhabdovirus Vesicular Stomatitis virus and rhinovirus, a *Picornaviridae* family member. The EC_50_ values of the active compounds are close to the 5 µM working concentration previously established with WP1130, a concentration, which also caused minimal toxicity in all analyzed cell lines and cell types. Compound-induced cellular toxicity in these compounds was not improved compared to the parent molecule WP1130, and further studies are needed to improve compound safety. However, these results establish the broad-spectrum antiviral properties of compounds **6** and **7**, while compounds **4** and **5** might be restricted in their antiviral activity to caliciviruses. Collectively, these data suggest that the WP1130 derivatives might target cellular molecules and/or processes that are required during the infectious cycle of multiple viruses from different virus families.

Targeting host proteins is a good strategy for inhibiting pathogens, as development of resistance to molecules that do not directly affect them is rare. Hence, molecules like WP1130, which target host proteins that modulate distinct cellular activities, may be useful templates for generating effective antivirals. WP1130 is a second-generation tyrphostin derivative (degrasyn) that acts as a partly selective deubiquitinase (DUB) inhibitor with known specificities for USP9x, USP5, USP14, UCHL-1 and UCH37 [37], [43]. Previous work demonstrates that the antiviral activity of WP1130 against MNV-1 is in part mediated by inhibition of the proteasome-associated DUB USP14 and activation of the unfolded protein response [35]. While additional target(s) of WP1130 and its active derivatives that mediate the antiviral effects remain to be identified, the interaction of WP1130 with DUBs is likely due to modification of catalytic-domain cysteine residues in select DUBs by adding to the β-position of the cyanoacrylamide in the WP1130 molecule. In support of this mechanism is the finding that removal of the cyano group (shown in compound **1**) or reduction of the double bond (shown in compound **2**) can render the WP1130 molecule “null” or inactive with regards to binding to its target DUBs or modulation of antiviral activity. Consistently, these “null” versions of the molecule showed no antiviral activity against any of the viruses tested.

Our previously published data [35] suggests that targeting of DUBs might enable development of more effective anti-norovirus or broader spectrum antiviral compounds. Thus chemical modifications in current DUB inhibitors, or synthesis of new compounds targeting DUBs, might prove useful in developing treatment options for the study of viruses *in vitro* and *in vivo*. Although WP1130 is quite effective as an antiviral, it suffers from low solubility and bioavailability [37], [38]. Therefore, the development of more soluble derivatives will enable future *in vivo* efficacy testing and will provide a means of identifying additional targets required by viruses for replication and potential new therapeutic targets. All active compounds examined in this study (compounds **4**–**7**) showed increased solubility compared to the parent compound. Solubility was calculated as per Lipinski et al. [44] for WP1130 (3.2 µM), and compounds **3** (19 µM), **5** (14 µM), **6** (160 µM), and **7** (14 µM). We could not calculate aqueous solubility due to limited availability for compounds **1**, **2**, and **4**, but we expect compounds **1** and **2** to have similar solubility to WP1130, and compound **4** to be more soluble than WP1130 but less soluble than compound **7**.

WP1130 has an aqueous kinetic solubility value of approximately 3.2 µM, with compounds **1**, **2** and **4** having similar values. With the exception of compound **4**, these small molecules were not active as antivirals in our screen. The addition of polar side chains increases solubility, which is predicted to result in increased bioavailability and target-compound interactions. Compounds **5**, **6** and **7** have predicted aqueous solubility values ranging from approximately 14 to 160 µM, which probably increased their uptake by host cells and the ability to reach their target molecules to exert the observed antiviral activities. The only exception is compound **3**, which has a polar side chain but had no effect on any of the viruses tested. It is possible that this particular side chain modification resulted in less significant interactions between DUB(s) and compound and/or in increased binding of other cellular proteins that are not necessary for viral replication. Interestingly, the highest antiviral activity was observed in compounds that, aside from being more soluble than WP1130, additionally have a pyridine or fluoropyridine function off the double bond rather than bromopyridine (compounds **5**–**7**). These structural modifications might allow the compounds to better interact with their target proteins, or be better absorbed by host cells.

Additionally, all effective compounds (including WP1130) retain USP9X inhibitory activity *in vitro* and *in vivo* to varying degrees (data not shown, N. Donato personal communication). Interestingly, WP1130 exhibited better antiviral activity in primary cells than any of its derivatives. This finding suggests differences exist between primary and transformed cells at the molecular level, e.g., with regards to the deubiquitinase profile. Future work will aim to elucidate the deubiquitinase modifications and profiles, as well as the mechanism for inhibition of viral replication to assist in cellular target identification and to further improve antiviral activity.

As WP1130 is a DUB inhibitor with antiviral activity [35], it is likely that its derivatives target similar molecules. In fact, compound **7** was shown to affect the levels of active DUBs when compared to untreated cells, suggesting it also inhibits one or more of these proteins. The ubiquitin pathway is often manipulated by viruses for their advantage [45], [46], and thus it is a good pathway to target for the development of therapeutics. This pathway involves the post-translational modification of proteins by the addition or removal of ubiquitin [47]. This modification affects proteins in many ways, ranging from signaling changes in the cell, substrate degradation, alterations in cellular localization and promotion or prevention of protein-protein interactions [47]. Viruses constantly take advantage of this pathway by modifying ubiquitination of cellular proteins, or their own proteins, to allow for increased replication efficacy [45], [46]. For example, DUBs such as USP9x and USP7 have been shown to aid in stabilization of proteins needed for EBV and HSV-1 infection [45], [48], [49], suggesting that their inhibition could provide ways to control or inhibit viral replication. Thus, targeting these DUBs with WP1130 or its derivatives could provide a way to counter a viral pathogen's attack on host cells.

In summary, herein we show the identification of small molecule derivatives of WP1130 with improved solubility and with broad spectrum antiviral activity *in vitro*. Although improved cellular toxicity was not yet achieved, these small molecules have the potential for further development into broad spectrum antiviral therapies. Identification of the DUBs or other antiviral cellular targets of these molecules might help to further improve the therapeutic potential of these compounds.
